# Socioeconomic and nutritional determinants outweigh gut microbiota influence on neurodevelopment in young children from Antananarivo, Madagascar

**DOI:** 10.1038/s41598-026-35174-5

**Published:** 2026-01-17

**Authors:** Jeanne Tamarelle, Maria V. Doria, Valérie Rambolamanana, Tatamo Rajaonarivo, Ana Sousa Ferreira, Maheninasy Rakotondrainipiana, Rindra Vatosoa Randremanana, Philippe Sansonetti, Pascale Vonaesch

**Affiliations:** 1 Department of Fundamental Microbiology, University of Lausanne, 1015 Lausanne, Switzerland; 2 Institut Pasteur, 25-28 Rue du Docteur Roux, Paris, France; 3 Unité d’Epidémiologie et de Recherche Clinique, Institut Pasteur de Madagascar, BP 1274, Ambatofotsikely, 101 Antananarivo, Madagascar; 4 Faculdade de Psicologia, Universidade de Lisboa, Alameda da Universidade, 1649-013 Lisbon, Portugal; 5 Business Research Unit (BRU-IUL), Instituto Universitário de Lisboa (ISCTE-IUL), Lisbon, Portugal; 6 Unité de Pathogénie Microbienne, Institut Pasteur, 25‑28 Rue du Dr Roux Paris, France; 7 Unité de Pathogénie Microbienne Moléculaire, Institut Pasteur, 25-28 Rue du Dr. Roux, 75015 Paris, France; 8 Hôpital Necker- Enfants Maladies, 149 rue de Sèvres, 75015 Paris, France; 9 Institut Pasteur de Madagascar, BP1274, Ambatofotsikely 101 Antananarivo, Madagascar; 10 Institut Pasteur, 25-28 Rue du Docteur Roux, Paris, France; 11 Biodiversity Research Center, University of British Columbia, 2212 Main Mall, Vancouver, Canada; 12 Institut Pasteur de Bangui, BP923, Bangui, Central African Republic; 13 Complexe Hospitalier Universitaire Pédiatrique de Bangui, Bangui, Central African Republic; 14 Hôpital Pitié-Salpêtrière, 47-83 Bd de l’Hôpital, 75013 Paris, France; 15 Centre Hospitalier Universitaire Mère Enfant de Tsaralalana, Antananarivo, Madagascar; 16 Institut Pasteur de Bangui, BP923, Bangui, Central African Republic

**Keywords:** Malnutrition, Microbiome, Clinical microbiology, Risk factors, Paediatric neurological disorders

## Abstract

In 2024, stunted child growth affected 150 million children under the age of five years, underscoring its critical impact on global health. Stunting has also been associated with neurodevelopmental delays. This study explores the relationship between stunting, the fecal microbiota, and neurodevelopment in 2–5-year-old children from the Afribiota cross-sectional study in Madagascar. Children were assessed using the Ages and Stages Questionnaire (ASQ-3), covering five developmental domains (communication, personal-social, problem-solving, fine and gross motor). Fecal samples were analyzed via 16S rRNA gene amplicon sequencing. Classical bi- and multivariate analysis was combined with Structural Equation Modelling to evaluate direct and indirect associations between different clinical factors, the microbiota and neurodevelopment. Our study shows that stunting and low socioeconomic status are consistently linked to poorer neurodevelopmental outcomes, while low branched-chain amino acids and hemoglobin levels are associated with stunting. Furthermore, a higher microbial diversity within individuals (α-diversity—specifically the Shannon index-) was directly linked to improved neurodevelopment scores in one of the tested models, while gut microbiota variation between individuals (β-diversity) was not associated with neurodevelopment. These findings support the hypothesis of neurodevelopment being primarily influenced by nutritional and social factors, with a more limited role for microbiota diversity.

**Supplementary Information:**

The online version contains supplementary material available at 10.1038/s41598-026-35174-5.

## Introduction

Stunting is a chronic form of childhood undernutrition and is often measured through the height for age Z-score (HAZ). Stunting is defined as children that are below two standard deviations from the median HAZ of the WHO reference^1^. The latest UNICEF/WHO/World Bank Joint Child Malnutrition Estimates report states that in 2024 there were 150 million children under the age of 5 years considered stunted. Thus, while there was a small improvement since 2000^2^, the number of affected children is still alarmingly high. Evidence has accumulated on the impact of early life stunting on brain development (reviewed in^3^). Of note, limited height cannot be causing delays in brain development per se^4^. When considered as a potential causal factor in the impairment of brain development, stunting rather serves as an indicator of multiple measurable and elusive underlying pathophysiological factors rather than just an anthropometric measure^5^. Prenatal and early childhood stunting can affect motor and neurodevelopment in the short-term, with effects visible as soon as at 2 years of age^6^ but being maintained during the first years of life^7,8^ and even into adulthood^9^. This in turn prevents children from reaching their full developmental potential, with effects such as late school enrolment and reduced educational achievements^10–13^ and subsequent income losses^14^. A study by Lu et al. estimated that, in 2010, there were still 249 million children under the age of 5 years at risk of poor development in low-and-middle income countries (LMICs)^15^. Other factors such as diet^16^, male sex, poverty, rural residence, lack of cognitive stimulation^17^, and anemia/iron deficiency (reviewed in^18^) are further contributing to poorer neurodevelopmental outcomes in children.

The etiology of stunting is complex and context dependent. Nevertheless, several factors including maternal factors (nutrition, education, pregnancies), birth weight and sanitation are consistently found to be associated with stunted child growth^19,20^. Previous research has shown that metabolites associated with stunting are partly shared with neurodevelopmental impairment^21^, making it difficult to disentangle the effects of the microbiota on undernutrition and neurodevelopment. Madagascar is one of the countries which is most affected by child stunting, with a national-level stunting prevalence in children under the age of 5 years of about 50%^22^. Previous studies carried out in Madagascar have shown that low maternal and paternal heights^23–27^, low maternal education level^27^, unspaced pregnancies^24^, low socioeconomic score^24^, low birth weight^23,24,27^, helminth infection^24^, low food diversity score^28^ and anemia^27,29^ are all associated with stunted child growth.

Enteric infections and diarrhea are more frequent in undernourished children and are at the same time also leading to a worsening of the nutritional state^30^. Diarrhea is also associated with impaired cognitive development^31^ and school performance^32^. Research within the context of the Afribiota study^33^ has shown a high prevalence of intestinal parasites^34^ and enteropathogens^35^ in the group of children studied.

Beside infectious factors, there is also a clear association between stunted child growth and the intestinal microbiota, with two key disease signatures: small intestinal overgrowth of bacteria of oral origin (termed SIOBO for small intestinal oral bacterial overgrowth) and a reduction in butyrate-producing strains in the feces^36,37^. In a hallmark study comparing germfree and conventional mice, it was clearly shown that the microbiota plays a key role in neurodevelopment^38^. Since then, numerous studies have shed light on the different mechanisms linking the intestinal microbiota, microbial metabolites and neurodevelopment. Most recently, in a mouse model where mice were overexposed to isolates from the small intestinal tract of stunted children, a clear causal link was established between SIOBO, local and systemic inflammation, and alterations in neurodevelopment and behavior^39^. However, disentangling the respective roles of enteropathogens, the microbiota, and stunting in child neurodevelopment remains challenging.

Here, our objective was to study the association between stunting, socioeconomic factors, the fecal microbiota and neurodevelopment in a cohort of 349 children aged 24–60 months in Madagascar. We focused our research on children older than 24 months, as previous research has shown that 70% of the deficit accumulated in height was due to faltering during the first 2 years of life^40^. In addition, the effects of chronic undernutrition on brain development might accumulate over time with stronger associations as children age^41^.

To address the complex interplay between these factors, we used a combination of classical bi- and multivariate models and a more complex structural equation modelling (SEM) approach. SEM is a multivariate statistical method, which relies on initial models built by the researcher and allows the simultaneous estimation of multiple regression equations, enabling the assessment of both direct and indirect (mediated) pathways while accounting for potential confounders. This approach is well suited to testing alternative conceptual frameworks, specifying potential directions of association between factors, and identifying the conceptual framework that best fits the observed data. We hypothesize that stunting is directly associated with lower neurodevelopmental scores, that socioeconomic factors and the fecal microbiota might either have a direct effect on neurodevelopment or an indirect effect through stunting.

## Results

### Description of household, maternal and child characteristics in the Afribiota subcohort

Three hundred forty-nine (349) children were included in this analysis, including 197 normally nourished (56.4%), 84 moderately stunted (24.1%) and 68 severely stunted children (19.5%). Sociodemographic characteristics of the households, mothers and children are displayed in Suppl Table 1. Sixteen and a half percent of the mothers of included children were undernourished, with different numbers per category of child stunting (13.0% for normally nourished children, 15.9% for moderately stunted children and 27.3% for severely stunted children, *p* = 0.026). Anemia was common in our study population (22.7%) and correlated with stunting severity (*p* = 0.035). On average children had introduced solid food at 5 months of age and ceased breastfeeding at 24 months of age, leaving only 8.3% of our study population currently breastfed at the time of the study. Sixty-six and a half percent of children had been exclusively breastfed for the first 6 months of age based on maternal recall. Twenty-eight point nine percent of children had a low dietary diversity, and 8.7% did not consume any animal source foods. Parasitic infections were frequent in our study population with infestation rates of 49.3%, 61.8% and 22.4% for *Ascaris*, *Trichuris* and *Giardia*, respectively.

### Child’s stunting is strongly associated with lower neurodevelopmental scores

Child stunting (HAZ score) was significantly associated with lower neurodevelopment scores in both unadjusted and adjusted linear regression models (adjusted for socioeconomic score, access to running water, child’s sex, child’s age, anemia, mother’s age at first pregnancy and mother’s educational level), with a gradient according to stunting severity (Table 1). Overall development scores ranged from 120 to 300 in our study, and in our adjusted models, moderately stunted children had a score on average − 10.2 points [95% CI − 17.7, − 2.7] lower than normally nourished children, while severely stunted children had a score on average − 18.6 points [− 26.6, − 10.6] lower. The communication (Comm) domain was not associated with stunting in adjusted models. The Personal-Social (PES) domain was only associated with moderate stunting. The Problem-Solving (PS), fine motor (FM) and gross motor (GM) domains were only associated with being severely stunted. PS score ranged from 10 to 60 in our study, and severe stunting was associated with a mean different of − 3.4 [− 6.5, − 0.2] points compared to normal growth. FM score ranged from 10 to 60 in our study, and severe stunting was associated with a mean difference of − 6.9 [− 9.8, − 4.0] points compared to normal growth. GM score ranged from 18 to 60 and severe stunting was associated with a mean difference of − 5.0 [− 7.3, − 2.8] points compared to normal growth.

**Table 1:** Mean difference in development score per domain and overall between normally-nourished children and moderately or severely stunted children (N = 349).

	Moderately stunted								Severly stunted							
	Unadjusted				Adjusted*				Unadjusted				Adjusted*			
	Mean difference		95% CI	*P*-value	Mean difference		95% CI	*P*-value	Mean difference		95% CI	*P*-value	Mean difference		95% CI	*P*-value
Overall score	− 12.4	− 19.8	− 5	0.001	− 10.2	− 17.7	− 2.7	0.008	− 20.9	− 28.9	− 12.9	0.000	− 18.6	− 26.6	− 10.6	0.000
Communication score	− 0.9	− 3	1.1	0.378	− 0.8	− 2.9	1.2	0.427	− 1.2	− 3.4	1	0.289	− 1.2	− 3.4	1.1	0.322
Problem-Solving score	− 3.5	− 6.6	− 0.5	0.024	− 2.4	− 5.3	0.6	0.115	− 3.6	− 6.9	− 0.3	0.031	− 3.4	− 6.5	− 0.2	0.038
Personal-Social score	− 2.8	− 5.2	− 0.5	0.017	− 3.1	− 5.4	− 0.8	0.008	− 2.2	− 4.8	0.3	0.080	− 2.1	− 4.5	0.4	0.099
Fine motor score	− 3.6	− 6.2	− 1	0.007	− 2.5	− 5.2	0.1	0.064	− 7.8	− 10.6	− 5	0.000	− 7	− 9.8	− 4.1	0.000
Gross motor score	− 1.5	− 3.7	0.7	0.192	− 1.3	− 3.4	0.8	0.234	− 6	− 8.4	− 3.6	0.000	− 5	− 7.2	− 2.7	0.000
Number of domains with delay	0.1	0	0.3	0.110	0.1	0	0.2	0.168	0.3	0.1	0.4	0.000	0.3	0.1	0.4	0.000

*Adjusted for socioeconomic score, access to running water, child’s sex, child’s age, anemia, mother’s age at first pregnancy and mother’s educational level.

Other factors associated with neurodevelopmental scores in each domain and with the overall neurodevelopmental score were investigated in bivariate analyses and multivariate analyses (Suppl Table 2). For the overall score and the FM score, only a higher HAZ score and higher maternal education remained significantly associated with a higher score in adjusted models. In the Comm domain, higher maternal education was positively associated with an increased score, while an older age and having an *Ascaris* infection were negatively associated. In the GM domain, an older age and a higher reported birth size were associated with an increased score. In the PS domain, having access to running water in the household, drinking treated water and a higher HAZ score were associated with an increased PS score, while an older age and having a *Blastocystis* infection were negatively associated with the PS score. Finally, in the PES domain, an older age and female sex were associated with increased score while detection of several enteropathogens’ genes in the feces was associated with a decreased score.

### Fecal microbiota composition is only marginally associated with neurodevelopment scores

**Fig. 1:**
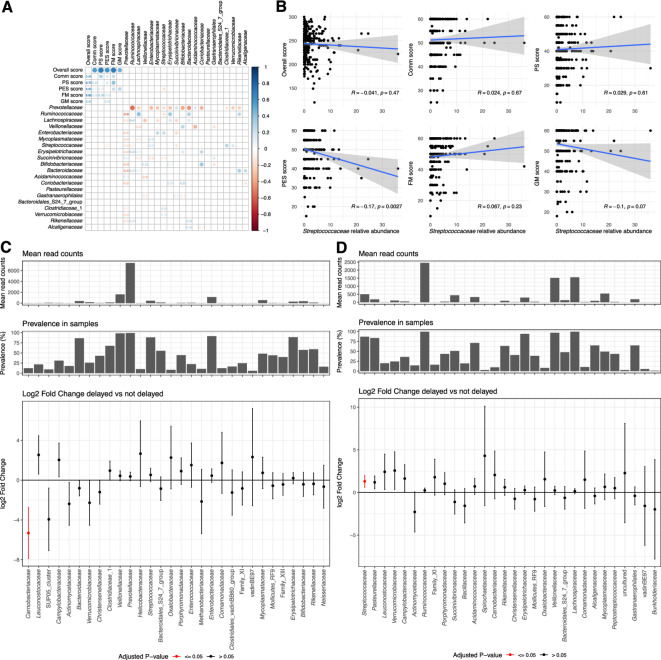
Association between individual bacterial genera and neurodevelopmental score. (**A**) Correlation matrix between each neurodevelopmental score and bacterial families. Only the top 20 families are displayed. *P*-values are corrected for multiple testing using the Benjamini–Hochberg correction. Only statistically significant results after correcting for multiple testing (q-value ≤ 0.05) are displayed. (**B**) Correlation between each neurodevelopmental domain and the *Streptococcaceae.* (**C**) and (**D**) DESeq2 restricted to the lowest and highest quartiles of the overall score distribution (**C**) and the Gross Motor score (**D**). Results are adjusted on sequencing run. *P*-values are corrected for multiple testing using the Benjamini–Hochberg correction. Results are ordered by increasing *p*-value and only the first 30 bacterial genera are displayed. PS: Problem-Solving, PES: Personal-Social; FM: Fine Motor; GM: Gross Motor; Comm: Communication.

For subsequent analyses, fecal microbiota data was available for 317 children. The dataset contained 1’626 amplicon sequencing variants (ASVs). Each sample had on average 22’039 16 s rRNA gene amplicon sequences (standard deviation 18’867). In terms of α-diversity, neither the richness (number of taxa) nor the Shannon and inverse Simpson indices (number and evenness of taxa) were associated with any of the developmental domains or with the overall neurodevelopmental score, except a significantly higher Shannon index associated with an increased FM score (Suppl Table 3). The data reduction approach based on hierarchical clustering generated two microbiota clusters (Suppl Figure 1). However, belonging to one of these two clusters was not associated with the developmental scores of children (Suppl Table 4). Furthermore, Principal Coordinate analysis (PCoA) using the Euclidian distance, the Bray–Curtis distance or the Aitchison distance explained only very little of the microbiota diversity (Suppl Figure 2). Correlations calculated between each domain score and the bacterial families revealed that one family, *Streptococcaceae*, was negatively associated with the PES score, after correcting for multiple testing (Fig. 1A, B). Another family, *Carnobacteriaceae*, was associated with the overall score using DESeq2, yet the prevalence and abundance of this family was low (Fig. 1C), making the association spurious. DESeq2 analyses also confirmed that *Streptococcaceae* was significantly associated with the PES score (Fig. 1D), although this result was not confirmed by ANCOM-BC (Suppl Figure 3). Other weak associations appeared, non-concordant between the DESeq2 and ANCOM-BC methods (Suppl Figure 4 and 5), at the genus and family level, and corresponding to very low abundance and low prevalence bacteria. Thus, for the following analyses, α-diversity represented by the Shannon index, relative abundance of *Streptococcaceae* (as this family correlated with the PES score and has also been shown previously to be associated with stunting^37,42,43^), the cluster variable as well as the first component of the PCoA based on the Euclidian distance on relative abundance data, which captured the highest amount of variability (Suppl Figure 2), were used alternatively as microbiota constructs in the SEM models to find the best fit to our data.

### Socioeconomic score and nutritional status directly contribute to neurodevelopmental score

**Fig. 2:**
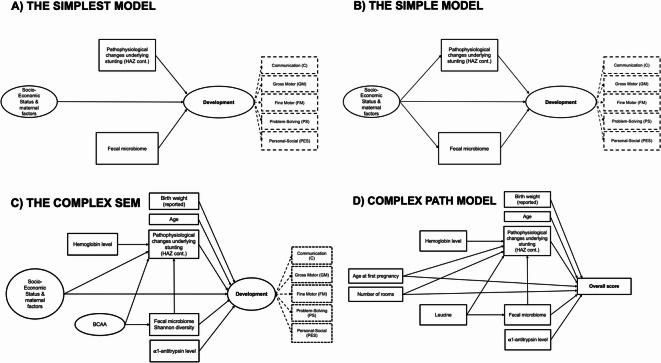
Tested conceptual models for the interplay between stunting, socioeconomic factors, the fecal microbiota and child neurodevelopment. (**A**) The simplest SEM model includes only direct effects of the socioeconomic status, the fecal microbiome and stunting on neurodevelopment; (**B**) the simple SEM model includes direct effects of the socioeconomic status, the fecal microbiome and stunting on neurodevelopment and an indirect effect of the socioeconomic status on neurodevelopment through stunting and through the fecal microbiome; (**C**) The complex SEM model includes the socioeconomic status, the fecal microbiome, stunting, anemia, BCAA, α1-antitrypsin level (AAT), age and reported birth size, allowing for direct and indirect effects on neurodevelopment; (**D**) The complex path model includes only observed variables (number of rooms, maternal age at first pregnancy, the fecal microbiome, stunting, anemia, leucine, α1-antitrypsin level, age and reported birth size), without latent constructs, allowing for direct and indirect effects on the overall developmental score. Observed variables are represented as squares and latent variables are represented as circles. Indicator variables used to build latent constructs are squared with dashed lines. HAZ: Height-for-Age Z-score; BCAA: branched-chain amino acids.

As linear regression models do not allow to test for both direct and indirect effects, we developed and tested four different conceptual frameworks for disentangling the direct and indirect roles of stunting, socioeconomic factors and the fecal microbiota on neurodevelopment (Fig. 2). In the three different SEM models tested (Fig. 2A–C), three latent variables were constructed based on correlation plots amongst indicator variables (Suppl Fig. 6). Confirmatory factor analysis indicated that modelling latent constructs for neurodevelopment, Socioeconomic Status and Maternal factors (SES), and branched-chain amino acids (BCAA) was fitting the data correctly (Suppl Table 5). Therefore, these latent variables were used further in the SEM models. The latent variable for neurodevelopment was based on the 5 per domain scores with the estimation of the model giving more weight to the PS and FM domains. The SES latent variable was based on the socioeconomic score, maternal education level, maternal age at first pregnancy, treated water as the main source of drinking water and number of rooms in the household, as these variables were most often associated with individual developmental domains scores (Suppl Table 2).

**Fig. 3:**
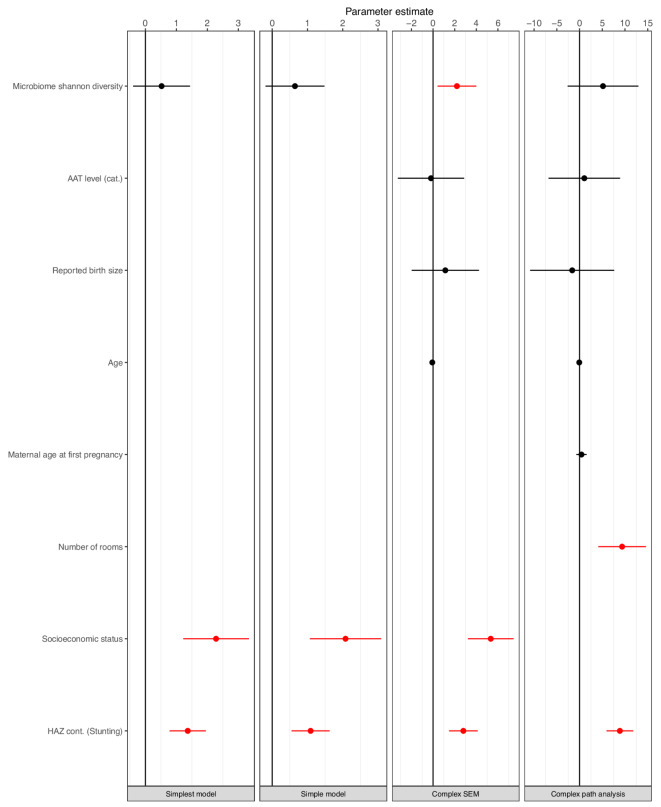
Mean direct effects (β-coefficients) of variables on neurodevelopment in the four different tested models: the simplest SEM, the simple SEM, the complex SEM and the complex path analysis. Only the results of the model using α-diversity Shannon index as microbiome construct are represented. Significant associations (*p*-value ≤ 0.05) are indicated in red. Units: AAT level: normal vs elevated; Reported birth size categories: Smaller than other babies, same as other babies, bigger than other babies; Age in months; Maternal age at first pregnancy in years. For AAT, values below 1.25 mg/g of fecal dry weight or below 0.15 of fecal wet weight were considered normal. AAT: α1-antitrypsin.

**Fig. 4:**
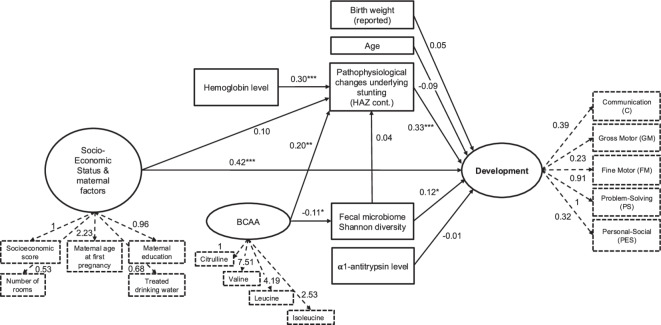
Theoretical framework including both the measurement model and the structural model, and standardized parameter estimates for direct mean effects in the complex structural equation modelling (SEM) model tested. The parameter estimates are standardized on all variables’ standard deviations, thus restricted between − 1 and 1. Observed variables are represented as squares and latent variables are represented as circles. Dashed arrows indicate the loadings of indicator variables in the measurement model (all significant). Each latent variable has one factor loading fixed to 1 for scaling purposes. Significant estimates are indicated with asterisks (*: ≤ 0.05, **: ≤ 0.01, ***: ≤ 0.001). HAZ: Height-for-Age Z-score; BCAA: branched-chain amino acids

All three SEM models were suiting the data well, whereas the fourth model, a path analysis model (without latent constructs), was not (Suppl Table 5). Whatever the model used, stunting was always significantly associated with neurodevelopment (as a latent variable in SEMs) or the overall neurodevelopmental score (for path analysis), with a higher HAZ score being associated with a higher development score (Suppl Table 6, Fig. 3 and Suppl Fig. 7). A higher SES was also significantly associated with a higher development score, with a direct effect, possibly driven by the variable “number of rooms in the household” (+ 9.2 mean difference in overall score for each additional room in the household in the path analysis model, *p* < 0.001). The indirect effect of the SES on neurodevelopment mediated by either the HAZ or the fecal microbiota was not significant in any of the tested models (Suppl Table 7). Age, reported birth size, maternal age at first pregnancy or α1-antitrypsin levels, a measure of protein-losing enteropathy often used as a biomarker of environmental enteric disorder (EED)^44^, were not associated with neurodevelopment (latent variable) in multivariate SEM or the overall score in path analysis. Finally, in the complex SEM model, a greater Shannon α-diversity was directly associated with higher neurodevelopment scores, without evidence of mediation through the pathophysiology underlying stunted growth. Other measures of fecal microbiota composition, such as *Streptococcaceae*, the first principal component of a PCoA or the two clusters resulting from hierarchical clustering were never associated with neurodevelopment in the tested SEMs nor with the overall score in path analysis. In addition, the indirect effect of the fecal microbiota on neurodevelopment (mediated by HAZ) was always non-significant, whatever the model used (Suppl Table 7). The standardized parameter estimates for direct mean effects for the complex SEM model (with Shannon diversity for the microbiota construct) are summarized in Fig. 4. Moreover, BCAA levels exerted an indirect effect on neurodevelopment mediated primarily through the pathophysiology associated with stunted growth and, to a lesser extent, through the fecal microbiome. Similarly, hemoglobin exhibited an indirect effect on neurodevelopment mediated by the pathophysiological processes linked to stunted growth. Of note, the model did not include a direct path from BCAAs and hemoglobin to neurodevelopment because in multivariate linear regressions these variables were not significantly associated with neurodevelopment.

## Discussion

In the present study, we aimed at estimating the direct and indirect effects of stunting, different socioeconomic factors and the fecal microbiota on neurodevelopment in children under the age of 5 years living in Madagascar. Beside classical bi- and multivariate analyses assessing for the association between given variables and microbial taxa and neurodevelopment, we used classical data reduction approaches in the microbiota field, such as PCoA, α-diversity indices or clustering methods to include the fecal microbiota in structural equation models (SEM).

Our study confirms the strong association between stunting measured through the HAZ score (and stunting severity) and lower neurodevelopment scores, in a per domain approach (personal-social, problem-solving, fine motor and gross motor) and a global score approach. We showed that stunting is associated with a lower overall score and with lower scores in the problem-solving, personal-social, fine motor and gross motor domains, using linear regression models, adjusting for important cofactors. Stunting (HAZ score) was a consistent predictor of neurodevelopment, even in more complex models. Here, it is important to note that stunting is measured through the HAZ score, an anthropometric measure that is unlikely to be a causal factor for impaired development per se. The HAZ score rather signals underlying multifactorial pathophysiological traits of chronic undernutrition that are difficult to capture^5^. Stunting has been previously associated with neurodevelopment, mostly in the cognition and motor domains. In the socioemotional domain, results are more contrasted due to poor comparability of socioemotional development measures. In longitudinal analyses, early onset of stunting and persistence until 5 years of age was particularly associated with poor development^45^, which confirms the hypothesis that the effect of stunting accumulates over time, and which might be why this association is visible even in cross-sectional analyses. Using linear regression models adjusting for important cofactors, we further demonstrate associations between neurodevelopmental scores (overall and per domain) and the presence of parasites and enteropathogens, sex, maternal education level, and markers of socioeconomic status such as access to running water and consumption of treated drinking water.

To go beyond direct associations, we then developed a theoretical framework including all variables associated with neurodevelopment in the multivariate models. We also included AAT as a key biomarker of environmental enteric disorder^44^. Using SEM and path analysis modelling, we then assessed for variables either directly or indirectly influencing neurodevelopment. We used a compositional measure (latent construct) of neurodevelopment based on each of the five domains evaluated in the Ages and Stages Questionnaires (ASQ-3) in several SEM models of increasing complexities and used the overall neurodevelopment score in a path analysis model. Of note, the SEM models showed a good fit with the data, while the path analysis model did not. In the SEM models, we show a direct effect of stunting and low socioeconomic status on impaired neurodevelopment, and no effect of age or reported birth weight. We also found no direct or indirect effect of α1-antitrypsin as a marker of overall intestinal inflammation and EED on neurodevelopment. Furthermore, we show a direct effect of low hemoglobin levels on stunting and an indirect effect on low neurodevelopment scores. High BCAA levels had a positive and direct influence on linear growth (HAZ score) and thus an indirect effect on neurodevelopment. Furthermore, BCAA levels were negatively associated with the Shannon diversity. Finally, Shannon diversity was directly and positively associated with increased neurodevelopment scores in the most complex SEM model.

In line with our results, socioeconomic status is a well-known predictor of neurodevelopment in children, although usually with small effect size^46,47^, and usually linked to language and communication abilities. In the above-mentioned study by Alam et al., socioeconomic status represented by the HME inventory and the WAMI index were positively associated with cognitive development at five years of age^45^. In another study from the MAL-ED network, socioeconomic status represented by environmental safety and healthfulness was directly positively associated with child development at 24 months of age using the BSID-III evaluation tool, and indirectly associated through hemoglobin concentration^48^. Our theoretical model differed slightly from the one proposed by the MAL-ED network. We included stunting (HAZ score) in the pathway between hemoglobin level and child development, as hemoglobin level was associated with stunting in the two countries of the Afribiota project^27^, leading to an indirect but positive association between hemoglobin level and child development.

The absence of effect of age on neurodevelopment is expected as the ASQ-3 tool is an age-specific test. The absence of association between neurodevelopment and reported birth size (which was well correlated with reported birth weight for children for whom we had access to this information) in the SEM models is more surprising. Of note, reported birth size was significantly associated in adjusted linear regression models only for the gross motor domain. In a previous study by Ranjitkar et al., using the Bayley Scales of Infant and Toddler development, 3rd edition (BSID-III), low birth weight was associated in Nepalese children aged 6–11 months old with a lower motor score, a lower cognitive score and a lower language score^49^. This was also the case in cohorts of infants born preterm or with very low birth weights^50–53^ or in meta-analysis^54^ using composite scales of development. However, similar to our findings, a previous study by Alam et al. on children followed until 5 years of age found no association between birth weight and cognitive development measured by the Wechsler Preschool Primary Scale of Intelligent III score, and no association between α1-antitrypsin and cognitive development either^45^.

We further included previously published amplicon sequencing data on the fecal microbiota in our analysis on neurodevelopment. Classical microbiota analysis assessing for α- and β-diversity as well as assessing for specific taxa associated with neurodevelopment showed only very weak associations between the microbiota and neurodevelopment. Furthermore, while it is technically feasible, testing all taxa individually in SEM models would inflate the probability of detecting false positive associations. We thus used several data reduction approaches (PCoA, clustering, α-diversity measures), which allow to account in addition for the compositionality of the microbiome data, in the SEM models. Using SEM approaches to disentangle the role of the fecal microbiota in undernutrition and neurodevelopment is not yet common and hampered by the high dimensionality of microbiota data. Lewis et al.^55^ and Tokuno et al.^56^ have defined microbiota latent variables based on some important taxa identified as significantly associated with the outcome in bivariate analyses. In our study, only few taxa were associated with neurodevelopment (although not robustly across tools). Therefore, this approach was not possible in our study. Vu et al*.* included key biomarkers of health and disease described in the literature such as the *Enterobacteriaceae* to *Bacteroidaceae* ratio or the presence of *C. difficile*^57^. Instead of focusing on individual taxa, we have chosen three different data reduction approaches that take into account the compositionality of microbiome data: the Shannon α-diversity index, the first component of a PCoA, and the partition into two groups of a hierarchical clustering approach^58^. Furthermore, seen the vast literature on the association of *Streptococcaceae* and undernutrition^37,42,43^, including previous work from the Afribiota consortium, we also included the *Streptococcaceae* family in the SEM models.

The microbiota is a complex entity, that can influence overall host physiology yet also is strongly shaped by it. A recent systematic review and meta-analysis by Chibuye et al. found no clear signature in terms of α-diversity (Shannon index) or β-diversity discriminating stunted and non-stunted children^59^. Nevertheless, recent research found a clear association of stunting with small intestinal oral bacterial overgrowth (SIOBO)^36,37^ and with a reduction in butyrate-producing taxa^36,37,59^. These changes are also directly implicated in pathophysiology including nutrient absorption^37^, inflammation^37^ and neurodevelopment^39^. Our data is compatible with a model where the direct role of the fecal microbiota on neurodevelopment is limited to α-diversity (measured by the Shannon index), with a modest effect size and not sustained in all models. A recent study by Portlock et al. assessed for links between moderate wasting, the fecal microbiota and brain development using multi-system Shapley additive value (SHAP) interpreted random forest models and network analysis. In the cohort of one-year-old children that they studied in Dhaka, Bangladesh, they found wasting to be significantly associated with alterations in neurodevelopment^43^. Furthermore, they confirmed the negative association between taxa usually found in the oral cavity but enriched in the feces, such as *Rothia mucilaginosa* and *Streptococcus salivarius,* and a depletion of *Bacteroides fragilis* in moderate wasting in this cohort. However, while we found similar microbiota changes^36,37^ to be associated with stunted child growth in the Afribiota study, we did not find a direct effect of *Streptococcaceae* on neurodevelopment.

The main limits of our study reside in its small sample size and its cross-sectional design. First, the Afribiota study was not designed to specifically perform SEM analyses, this is a secondary data use analysis. Thus, our limited sample size only allowed for moderately complex models in the SEM approach, as the number of parameters to estimate easily increases with the complexity of the model. Including several variables related to the same block was possible for some latent constructs but not all. Second, parasites and enteropathogens detection were not correlated enough to be able to build a latent construct, and it was not possible to include all these variables separately in the models, as this would have increased the number of parameters to estimate to unreasonable levels. On the association between enteropathogens, illness, hemoglobin and cognitive scores, we point to the study from the MAL-ED network investigators that used a similar SEM approach^48^. Another limit resides in the fact that psychologists were not blinded to the nutritional status of the evaluated children, which could then introduce a bias in the neurodevelopment evaluation. Finally, the ability of the microbiota constructs used in the different models to capture the complexity of the fecal microbiota is limited. Indeed, the Shannon index for α-diversity is but one index, representing both the richness and evenness of taxa in our samples, but widely different microbiota compositions can have similar diversity indices. The first component of a PCoA based on Euclidean distances matrix only explained 18% of the variance observed in our microbiota data. Finally, clustering methods might be subject to biased choices regarding the taxonomic level, the distance metrics, the clustering methods, and in the field of the gut microbiota they have yielded equivocal results^60^. Last, the use of SEM models could be greatly improved by longitudinal information, to infer causality links between variables. Future studies should further include additional information on metabolites, metagenomic and metatranscriptomics profiles to allow for more precise associations between bacterial taxa, metabolites and neurodevelopmental outcomes.

Nevertheless, our study has several strengths: to date, there are very few studies that performed SEM analysis to disentangle different factors that could contribute directly or indirectly to a disease outcome, which is important especially in syndromes such as stunting, that are multifaceted. We analyzed data from the Afribiota study using linear regressions, structural equation modeling (SEM), and path analysis. While linear regression models assess associations between specific variables and neurodevelopment, SEM offers key advantages: it incorporates latent constructs, estimates both direct and indirect effects, and accounts for multiple directional pathways within a unified framework. Finally, we provide data on a region where there is only scarce data to date on both early childhood neurodevelopment and the microbiota.

Our analyses showed that the association between microbiome diversity and neurodevelopment emerged only in the most complex SEM and represented a direct effect, independent of the pathophysiological processes underlying stunting. These results indicate that the fecal microbiome and stunting exert independent influences on neurodevelopment, with nutritional factors playing a predominant role. If confirmed in larger longitudinal studies, these findings suggest that microbiome-targeted interventions alone may be insufficient to improve neurodevelopmental outcomes without concurrent nutritional support.

## Conclusion

Our study establishes an association between stunting, socioeconomic status, and neurodevelopmental performance, as evaluated by the ASQ-3 screening instrument, among children aged 2–5 years living in Madagascar. Through the application of multiple statistical modeling approaches, the findings indicate a direct and significant effect of socioeconomic status on neurodevelopmental outcomes, a modest direct association between gut microbiome α-diversity and neurodevelopment, and no evidence supporting an indirect pathway of the microbiome mediated by the pathophysiological mechanisms underlying stunted child growth. These results highlight the predominance of socioeconomic determinants in shaping early neurodevelopment within our study population. Future investigations should employ a longitudinal design to elucidate temporal relationships among stunting, gut microbiota composition and neurodevelopment, allowing to build a comprehensive causal framework describing their interconnections during early child development.

## Methods

### Study design and population

#### The Afribiota study in Madagascar

The Afribiota study is a case–control cross-sectional study carried out between December 2016 and May 2018 in Madagascar and the Central African Republic, on stunted and non-stunted children aged 24–60 months. Stunting has been defined based on the height-for-age Z-score (HAZ) according to the WHO reference population^61^ with a cut-off of ≤− 2 standard deviations (SD) below the median defined as stunted growth and ≤− 3 SD defined as severely stunted growth. The study protocol has been published previously^33^. Briefly, children were recruited based on the following inclusion criteria: HIV-negative, not suffering from acute undernutrition (wasting, weight-for-height Z-score (WHZ) ≤ − 2 SD according to the WHO reference cohort^61^) or any other severe disease, living in two neighborhoods of Antananarivo (Ankasina or Andranomanalina Isotry). Included children were admitted to the hospital for sample collection and anthropometric measurements. Stunted children were matched with control children (HAZ > 2 SD) according to age (24–35 months, 36–47 months and 48–60 months), sex, neighborhood (same neighborhood or adjacent neighborhood), and season of inclusion (dry or wet season), as described previously^33^. In Madagascar, children were also evaluated regarding their neurocognitive development.

#### Stunting and neurodevelopment measurements

For the HAZ score, height was measured by trained personnel to the nearest 0.1 cm in a standing position using collapsible height boards (ShorrBoard Measuring Board, Maryland, USA). Weight was measured to the nearest 100 g using a weighing scale (KERN, ref. MGB 150K100 and EKS, Inter-équipement Madagascar).

Neurocognitive development was assessed using a translated and adapted version of the Age and Stages Questionnaires (ASQ) screening test^62,63^ in its third edition^64^. It consists of a questionnaire for each of the defined intervals for the following ages: 24, 27, 30, 33, 36, 42, 48, 54, and 60 months old, containing questions about simple tasks performed by the child in each of the following 5 areas: communication, fine motor (FM), gross motor (GM), Problem-Solving (PS), Personal-Social (PES). Trained psychologists administered the test by both observing the performance of the child on several tasks and by collecting the answers of the parents^65^. An overall development score was calculated as the sum of all five domains and used in path analysis (see below). The five domains were also used to compute a latent construct labelled “neurodevelopment” with each domain having different weights (loadings) in the “neurodevelopment” latent variable (Fig. 4). No cut-offs for defining on-track development or delay were used, as these have not been validated for Malagasy children. The ASQ-3 test was carried out in a separate visit, 12 days before the main visit on average (range [− 47, 53]).

#### Home, maternal and child variables

Household/family, maternal and child additional variables were collected using a standardized paper questionnaire in French, translated to Malagasy by the staff. A socioeconomic score was defined as previously described^27^. In short, the socioeconomic score was based on a minimal set of assets, including housing materials (floor and wall materials, ownership of a car, telephone, bike, motorcycle), access to specific utilities (electricity, bathroom, cooking location), and family size. These assets led to grouping into three distinct clusters in a principal component analysis that were checked using iteration tests^27^. The categories were then defined as follows: (1) lowest socioeconomic score: no telephone or house floor made of pounded earth; (2) middle socioeconomic score: telephone, wooden or concrete house floor, no internal shower or internal kitchen; (3) highest score: telephone, wooden or concrete house floor, either an internal shower or internal kitchen (separate room). We calculated a Dietary Diversity Score (DDS) consisting of a score ranging from 0 to 7 defined as the sum of the number of food groups consumed by the child in the last 24 h^66,67^. The 7 different food groups were: (1) grains, roots, tubers and plantains, (2) pulses (beans, peas, lentils), nuts and seeds, (3) dairy products (milk, infant formula, yogurt, cheese), (4) flesh foods (meat, fish, poultry, organ meats), (5) eggs, (6) vitamin-A rich fruits and vegetables; and (7) other fruits and vegetables. A Low Dietary Diversity (LDD) was defined as having a DDS strictly below 4. We also included a variable on animal source food consumed by the child in the last 24 h, including eggs, meat, fish, dairy products.

#### Biological markers

Biological markers were measured on the blood collected, as previously described^44^: Ferritin was measured and corrected for systemic inflammation^68^, using a correction factor of 0.67 if the C-reactive protein (CRP) level was above 6 mg/L. A low ferritin level was defined as below 12 μg/L. Hemoglobin values were adjusted for altitude^69^ and anemia was defined as a hemoglobin level below 11 g/dL^29^. CRP level was used as an inflammation marker, and considered elevated if above 10 mg/L. The following branched-chain amino acids (BCAA) were also measured as described previously^44^: Alanine, Citrulline, Valine, Leucine and Isoleucine. Citrulline was considered low if below 7 μmol/L and elevated if above 43 μmol/L.

Bacterial pathogen load was determined using quantitative PCR on a set of bacterial virulence genes (*ompC, ipaH, estla, eltB, eae, bfpA, aggR, aaiC, cadF, ctxA*) as published previously^35^. Presence of a given gene was based on a Ct value < 37, and the total number of genes present in each fecal sample was reported. Helminths (*Ascaris, Trichuris, Enterobius* and *Hymenolepis*) and Protozoa (*Giardia, Blastocystis*) were detected using the Merthiolate-Iodine-Formaldehyde technique and the Kato-Katz technique as published previously^34^.

#### Microbiota characterization methods

As described previously^37^, fecal samples were collected at the inclusion visit, aliquoted on site and directly snap-frozen in liquid nitrogen and then transferred to a − 80 °C freezer. DNA extraction was performed using commercial kits (QiaAmp cador Pathogen Mini or cador Pathogen 96 QIAcube HT Kit; Qiagen) following the manufacturer’s recommendations, with an additional bead-beating step. Library preparation and sequencing were carried out by Microbiome Insights. The primers used for library preparation were v4.SA501–v4.SA508 and v4.SA701–v4.SA712, as recommended by Kozich et al.^70^ and used within the framework of the Earth Microbiome Project. Numerous prior studies on the human microbiota have employed the same standardized primer set, thus facilitating cross-comparison of our data to other datasets. Sequencing was then carried out on an Illumina’s MiSeq platform, using the MiSEq 500 Cycle V2 Reagent Kit (250 × 2). Demultiplexed reads were processed using the dada2 pipeline^71^ and taxonomy was assigned using the Silva Reference Database (version 128) as described previously^37^.

### Statistical analysis

#### Bivariate and multivariate regressions

All analyses were carried out on R Statistical Software (version 4.1.2, R Core Team 2021)^72^. We first investigated the association between nutritional status and neurodevelopment scores for each development domain and overall using bivariate and multivariate linear regression models. This allowed both to confirm previously published results from the literature and to identify variables associated with nutritional status and neurodevelopment scores to be included in our SEM models (see below). The associations between household characteristics, maternal factors, socioeconomic status, biological markers, parasitic infections and dietary factors and (i) stunting status or (ii) development scores were therefore computed.

- For variables associated with stunting status (i), absolute numbers and percentages were given in each category of the outcome (normally nourished, stunted, severely stunted) for binary and categorical variables, and the Fisher’s exact test was used for statistical significance. Means (minimum–maximum) were given in each category of the outcome for continuous variables, and the Kruskal–Wallis rank sum test or the Pearson’s Chi-squared tests were used for statistical significance.
- For variables associated with development scores (ii), variables statistically significant in bivariate linear regression models were used to build multivariate linear regression models, excluding collinear or redundant variables, using the *lm* function of the *lmtest* package^73^ (version 0.9.40).
- To specifically assess the association between stunting (HAZ score in categories) and each neurodevelopment score, we built multivariate models adjusted for socioeconomic score, access to running water, child’s sex, child’s age, anemia, mother’s age at first pregnancy and mother’s educational level as these are factors identified in the literature to be associated with stunting and neurodevelopment (see introduction).

For each multivariate model, the assumptions were verified graphically using diagnostic plots (function plot(model) in the in the base library): Residuals vs Fitted plot for the linear relationship assumption; normal Q-Q plot for the normality of the distribution of residuals; Scale-Location plot for the homoscedasticity of the variance of residuals; Residuals vs Leverage plot for identifying influential cases or extreme values. In addition, the Breusch–Pagan test was used to check homoskedasticity in bivariable and multivariable linear regression models, using the function *bptest* in the *lmtest* package (version 0.9.40).

#### Microbiota analyses

The association between the fecal microbiota and neurodevelopmental domains and overall neurodevelopmental score was performed using several data reduction approaches. This was a preliminary step to determine which microbiota construct to include in the SEM models (see below).

α-diversity was evaluated on unaggregated reads (Amplicon Sequencing Variants level), without prior rarefaction, using the Shannon index and the inverse Simpson index in the *diversity* function of the *vegan* package^74^ (version 2.6.4), as well as the richness in the *estimate* function of the *vegan* package. Linear regression models were run on each of these indices (exposures) and neurodevelopment scores (outcome), adjusting for sequencing run and the β coefficient, the *p*-value and the R^2^ were reported.

Associations between individual taxa and neurodevelopment scores were evaluated at the family and genus levels using relative abundances. Correlations between individual bacterial families and genera and neurodevelopment scores were calculated using Pearson’ r. The results were corrected for multiple testing using the Benjamini–Hochberg correction. We also performed differential abundance testing at the family and genus level using the packages *ANCOM-BC*^75^ (version 1.4.0) and *DESeq2*^76^ (version 1.34.0). DESeq2 is known to be less stringent than ANCOM-BC^77^. Because no cut-offs adapted to Malagasy children have been proposed so far, we used the first and last quartiles of the distribution of each continuous score as a binary covariate in order to identify only strong signals to be included in the SEM models. These analyses were adjusted for sequencing run and corrected for multiple testing using the Benjamini–Hochberg correction.

β-diversity was evaluated using Principal Coordinate Analysis (PCoA). Distances between samples were calculated using alternatively (i) the Euclidean distance on relative abundance of reads, (ii) the Bray–Curtis distance on relative abundance of reads, (iii) the Euclidean distance on centered log-ratio transformed data (Aitchison distance). The resulting plots are presented in Suppl Fig. 2. For hierarchical clustering, distances between samples were calculated using the Euclidean distance on relative abundance of reads and the Ward linkage, using the option “Ward.D2” in the *hclust* function. The number of clusters was determined visually using the elbow method^78^ based on the Calinski-Harabasz index and the *clusterCrit* package (version 1.2.8)—Suppl Fig. 1. The association between clusters and each developmental score was assessed by Wilcoxon rank sum test for bivariate analysis and ANOVA for bivariate analysis adjusted for sequencing run.

#### SEM and path analysis conceptual framework

Finally, we built a theoretical framework of the relationships between stunting, fecal microbiota composition, neurodevelopment (main outcome) and other related dimensions before applying SEM and path analysis. SEM models or path analysis are multivariate statistical methods that allow modelling complex relationships between variables. In contrast to multivariate regressions, they further allow to model direct and indirect effects, i.e. hypothesizing on the directionality of associations. From the model equations, these models derive the model-implied covariance matrix and compares it to the real data variance–covariance matrix, trying to find the parameters of the model that minimize the difference. SEM differs from path analysis by combining this with factor analysis, allowing for the construction of latent variables based on observed variables known as indicator variables. Path analysis only allows for observed variables to be included in the model.

The main outcome was child neurodevelopment. Stunting was integrated in the SEM using the HAZ score (continuous variable).

We investigated several alternative models represented in Fig. 2:

i. The simplest SEM possible including socioeconomic status, stunting, fecal microbiota composition and neurodevelopment, without indirect effects;
ii. A simple SEM including socioeconomic status, stunting, fecal microbiota composition and neurodevelopment, allowing for a direct and indirect effects of socioeconomic status on neurodevelopment (through stunting and through the fecal microbiota);
iii. A complex SEM including socioeconomic status, stunting, fecal microbiota composition, neurodevelopment and other related dimensions listed below, allowing for direct and indirect effects;
iv. A complex path model including socioeconomic variables, stunting, fecal microbiota composition, neurodevelopment and other related dimensions listed below, allowing for direct and indirect effects, but without latent constructs (*i.e.*, using only observed variables).

#### Construction of latent variables for the SEM models

SEM combines regression analysis with factor analysis (latent/unobserved variables), while path analysis does not include factor analysis. Hence, both analyses require to build a structural model as shown in Fig. 2, but SEM models additionally require a measurement model for latent variables.

The different dimensions contributing to child neurodevelopment were grouped by blocks: socio-economic status and maternal factors, anemia, stunting, age, birth size, microbiota, BCAA and inflammation. In each of these blocks, several variables were considered. For (i), (ii) and (iii), we first determined latent constructs for the measurement model by calculating pairwise correlations among variables of a block. If variables were continuous, we used Spearman’s r coefficient. If they were categorical, we used Cramer’s V statistic, and if one was continuous and the other one categorical, we used the square root of the R2 of an ANOVA test. By visual inspection of the correlation plots, it became obvious that only three blocks were susceptible to be used for latent constructs: neurodevelopment, socioeconomic status and maternal factors and BCAA. All the other blocks with at least 4 variables had too low correlations (< 0.2) to build latent constructs. We then applied confirmatory factor analysis implemented in the *lavaan* R package^79^ (version 0.6.12) to confirm that only these three blocks could be transformed into latent constructs. The selection of observed and latent variables to include in the measurement model was based on the following criteria: (1) at least 4 indicator variables to determine a latent construct to allow identification of the model (i.e. enough degrees of freedom to estimate the parameters and test the fitness of the model to the data), (2) if less than 4 indicator variables, replacement of the latent construct by a variable for which the association with endogenous variables is the strongest. Latent variables are indicated as circles and observed variables as rectangles in the different models represented in Figs. 2 and 4.

#### Structural models for SEM and path analysis

For microbiota data, given the high dimensionality of the data, it was not possible to include all taxa in the model to build a microbiota latent construct, therefore we used four alternative approaches to include the relevant variables in each of the four models:

- using the first component of a PCoA using Euclidean distances on relative abundance data. We chose this method because it generated a first component that explained up to 18% of the variance of the microbiota dataset (Suppl Fig. 2), while the other methods explained less (Bray–Curtis or Aitchison);
- using the clusters issued from the hierarchical clustering procedure described above;
- using the widely used α-diversity Shannon index;
- using the *Streptococcaceae* family as it showed a significant association with a neurodevelopmental domain and has been previously associated with stunting^37,42,43^.

For each model (SEM or path analysis), we separately tested each of these four microbiota constructs and reported all results.

For the structural model, the main outcome was neurodevelopment, inserted in the model either as a latent variable for SEM models (i, ii, iii) or as the overall neurodevelopment score for path analysis (iv). The selection of variables for the structural model of SEMs and path models was based on the following parsimonious criteria: (1) previously demonstrated association with child development and/or stunting in bivariate analysis, (2) low rate of missing data. We then applied SEM using the *sem* function of the *lavaan* package^79^ (version 0.6.12). This function was also used for path analysis (iv), without specifying a measurement model. The fit of the different tested models was assessed in several ways (Suppl Table 5): a chi-square test comparing the model-implied covariance matrix to the real data variance–covariance matrix; the Comparative Fit Index (CFI) that compares how well the model fits the data compared to a null model; the Ticker-Lewis Index (TLI), similar to the CFI but penalizing for the number of parameters; the Root Mean Square Error of Approximation (RMSEA) that measures the discrepancy between the model and the data per degree of freedom; and the Standardized Root Mean Square Residual (SRMR) that measures the standardized difference between the observed and the predicted correlations.

## Supplementary Information

Supplementary Information 1.

Supplementary Information 2.

Supplementary Information 3.

Supplementary Information 4.

Supplementary Information 5.

Supplementary Information 6.

Supplementary Information 7.

Supplementary Information 8.

## Data Availability

All metataxonomic data are deposited on ENA ([https://www.ebi.ac.uk/ena/browser/view/PRJEB48119](https:/www.ebi.ac.uk/ena/browser/view/PRJEB48119)). Access to the remaining data is regulated through a Scientific Advisory Board, under the supervision of Institut Pasteur. Data requests should be directed to the corresponding author (PV), who will redirect them to the Access Committee.
