# Supplementary material for: Socioeconomic and nutritional determinants outweigh gut microbiota influence on neurodevelopment in young children from Antananarivo, Madagascar
Source: Sci Rep. 2026 Jan 17;16:5484. doi: 10.1038/s41598-026-35174-5 (PMC12886980; doi:10.1038/s41598-026-35174-5)
Supplement: Supplementary file 1 — Supplementary Information 1. [file 41598_2026_35174_MOESM1_ESM.docx]

**Socioeconomic and Nutritional Determinants Outweigh Gut Microbiota Influence on Neurodevelopment in Young Children from Antananarivo, Madagascar**

Jeanne Tamarelle^$ 1^, Maria V. Doria^$^ * ^2^, Valérie Rambolamanana ^3^, Ana Sousa Ferreira ^4, 5^, Maheninasy Rakotondrainipiana ^3^, Rindra Vatosoa Randremanana ^3^, Philippe Sansonetti ^6^, Pascale Vonaesch* ^1^on behalf of the Afribiota investigators

* Correspondence to: [pascale.vonaesch@unil.ch](mailto:pascale.vonaesch@unil.ch); mdoria@ymail.com

^$^ Contributed equally

^1^ Department of Fundamental Microbiology, University of Lausanne

1015 Lausanne, Switzerland

^2^ Institut Pasteur de Paris

25-28 rue du Docteur Roux, Paris, France

^3^ Unité d’Epidémiologie et de Recherche Clinique, Institut Pasteur de Madagascar

BP 1274, Ambatofotsikely, 101 Antananarivo, Madagascar

^4^ Faculdade de Psicologia, Universidade de Lisboa

Alameda da Universidade, 1649-013 Lisboa, Portugal

^5^ Business Research Unit (BRU- IUL), Instituto Universitário de Lisboa (ISCTE-IUL)

Lisboa, Portugal

^6^ Unité de Pathogénie Microbienne, Institut Pasteur

25‑28 Rue du Dr Roux, Paris, France

**SUPPLEMENTARY FIGURES**

**SUPPL FIGURE 1.** Heatmap of the 20 most abundant bacterial amplicon sequencing variants’ relative abundance after ascending agglomerative hierarchical clustering of participants based on the Bray-Curtis distance and Ward linkage. The hierarchical clustering resulted in two community clusters (1 and 2) used in subsequent analyses.

Reads counts are in relative abundance.

HAZ: Height-for-Age Z-score.

**SUPPL FIGURE 2.** Principal Coordinates Analysis (PCoA) of 317 fecal samples after data reduction of bacterial amplicon sequencing variants into two dimensions, using alternatively A) Euclidian distance on relative abundance data, B) Bray-Curtis distance on relative abundance data, and C) Aitchison distance, i.e. Euclidian distance on centered log-ratio (CLR) transformed data. The percentages on each axis indicate the percentage of variance explained by the principal component.

**SUPPL FIGURE 3.** Association between individual bacterial genera and neurodevelopmental score. A) Correlation matrix between each neurodevelopmental score and bacterial families. Only the top 20 families are displayed. P-values are corrected for multiple testing using the Benjamini-Hochberg correction. Only statistically significant results after correcting for multiple testing (q-value ≤ 0.05) are displayed. B) Correlation between each neurodevelopmental domain and the *Streptococcaceae.* C) and D) DESeq2 restricted to the lowest and highest quartiles of the overall score distribution (C) and the Gross Motor score (D). Results are adjusted on sequencing run. P-values are corrected for multiple testing using the Benjamini-Hochberg correction. Results are ordered by increasing p-value and only the first 30 bacterial genera are displayed.

PS: Problem-Solving, PES: Personal-Social; FM: Fine Motor; GM: Gross Motor; Comm: Communication.

**SUPPL FIGURE 4.** Association between individual bacterial families using ANCOM-BC in the lowest and highest quartiles of the score distribution and A) the overall score, B) the Personal-Social score.

Results are adjusted on sequencing run. P-values are corrected for multiple testing using the Benjamini-Hochberg correction. Results are ordered by increasing p-value and only the first 30 bacterial families are displayed.

**SUPPL FIGURE 5.** Association between individual bacterial genera using ANCOM-BC in the lowest and highest quartiles of the score distribution and A) the overall score, B) the Gross Motor score.

Results are adjusted on sequencing run. P-values are corrected for multiple testing using the Benjamini-Hochberg correction. Results are ordered by increasing p-value and only the first 30 bacterial families are displayed.

**A)**


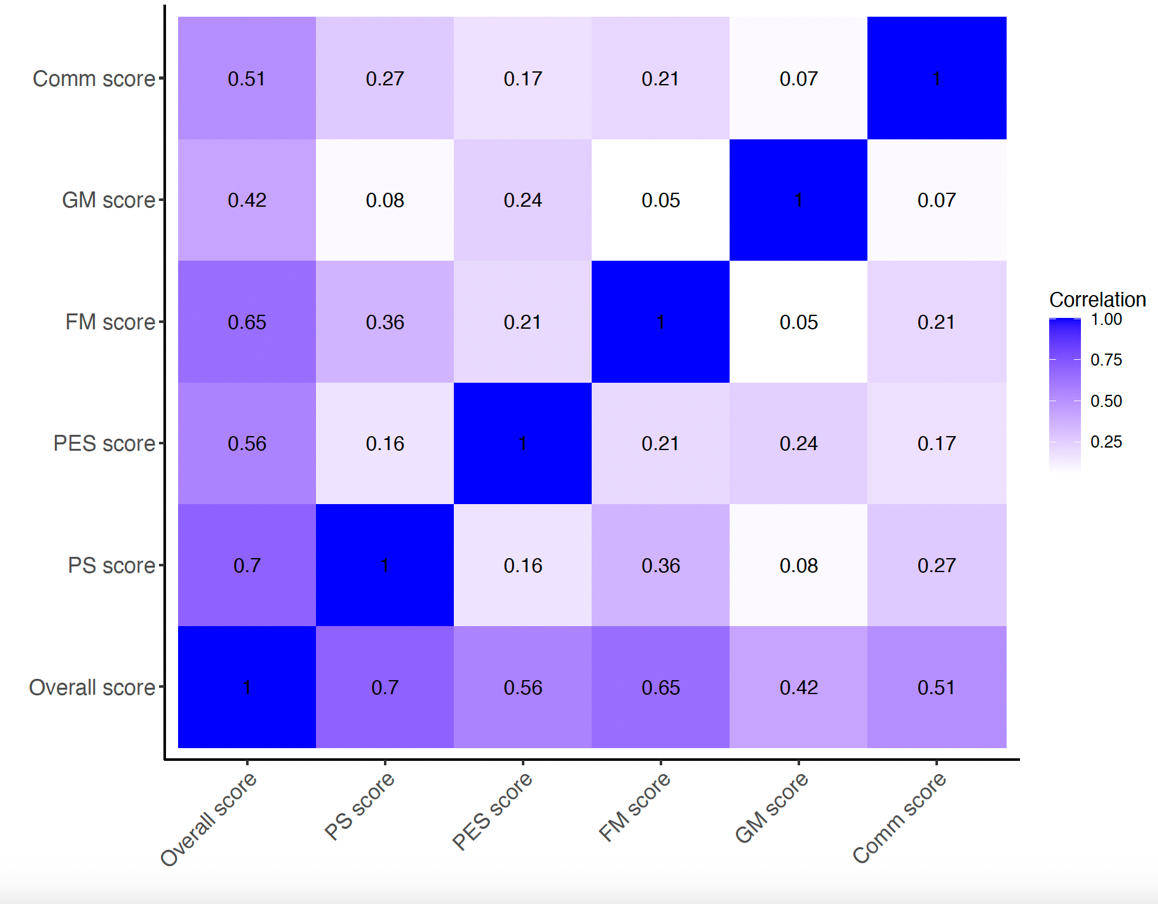


**B)**

**
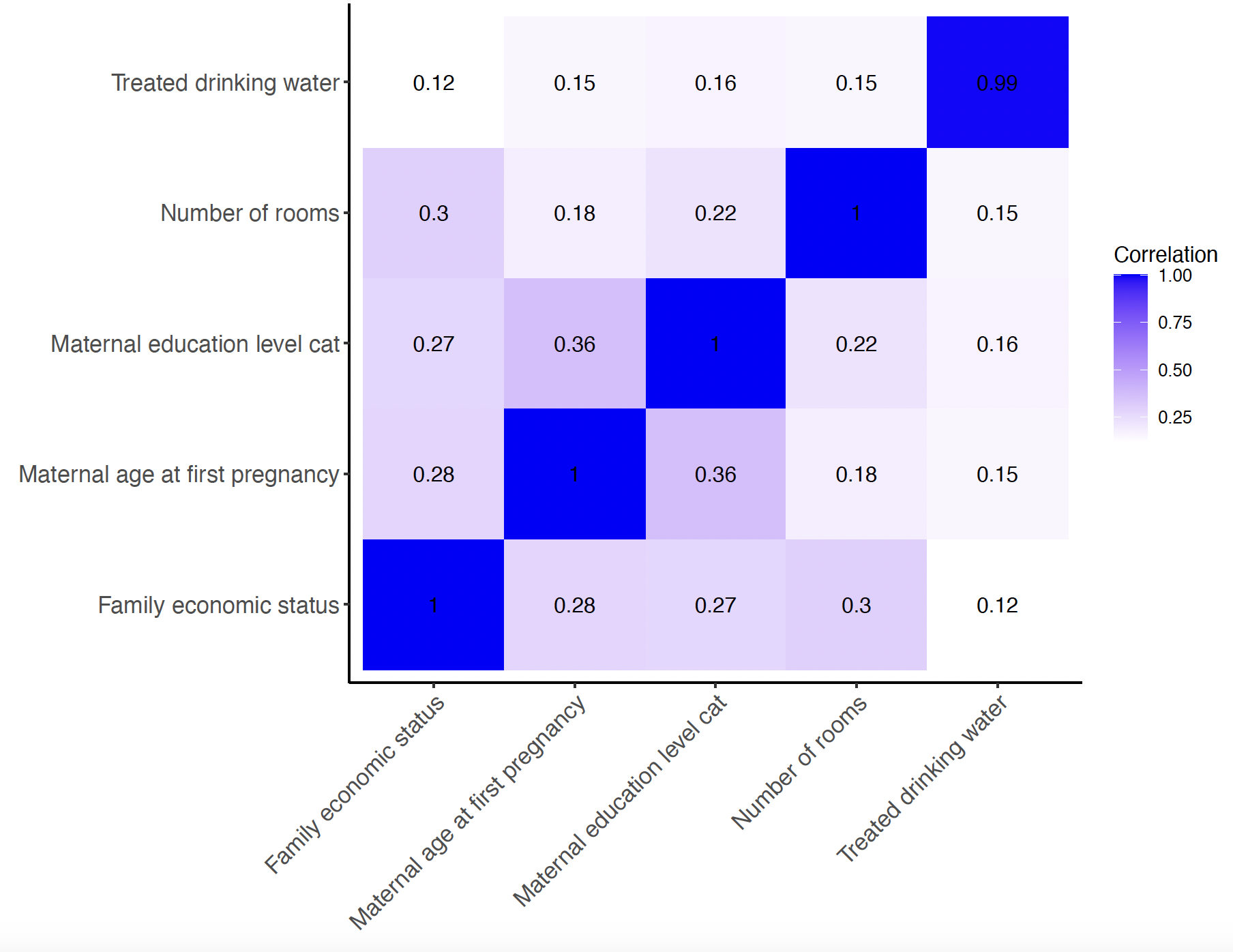
**

**C)**

**
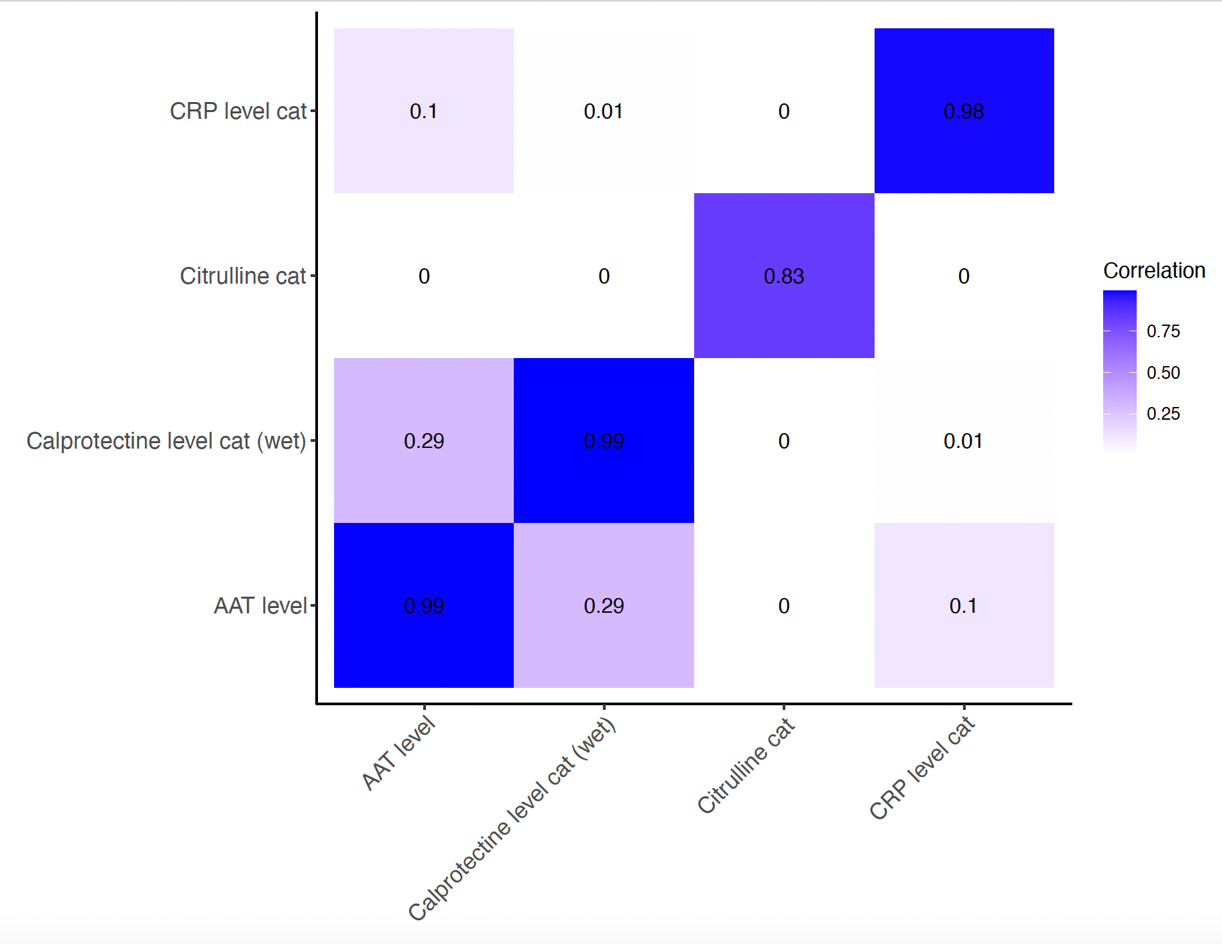
**

**D)**

**
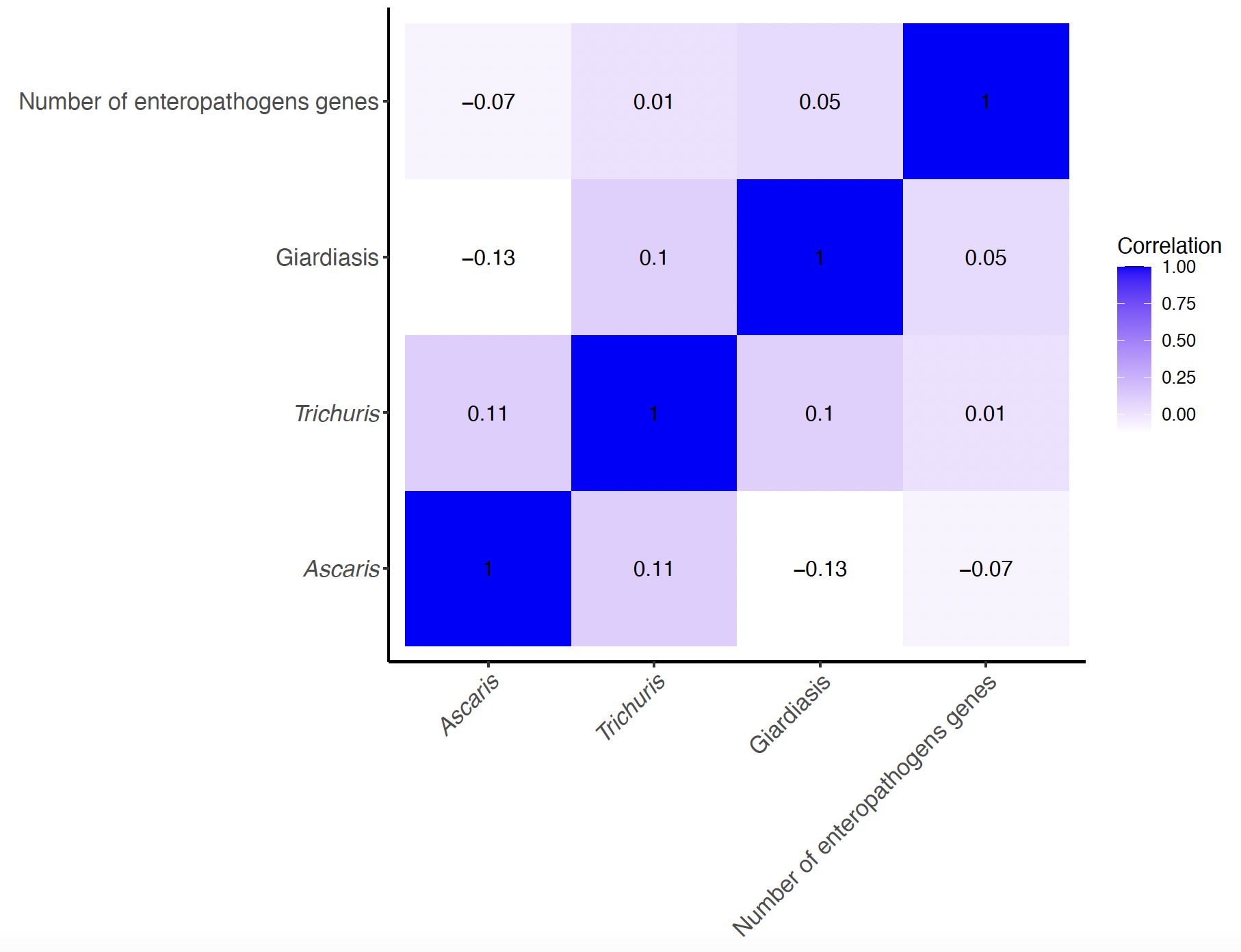
**

**E)**

**
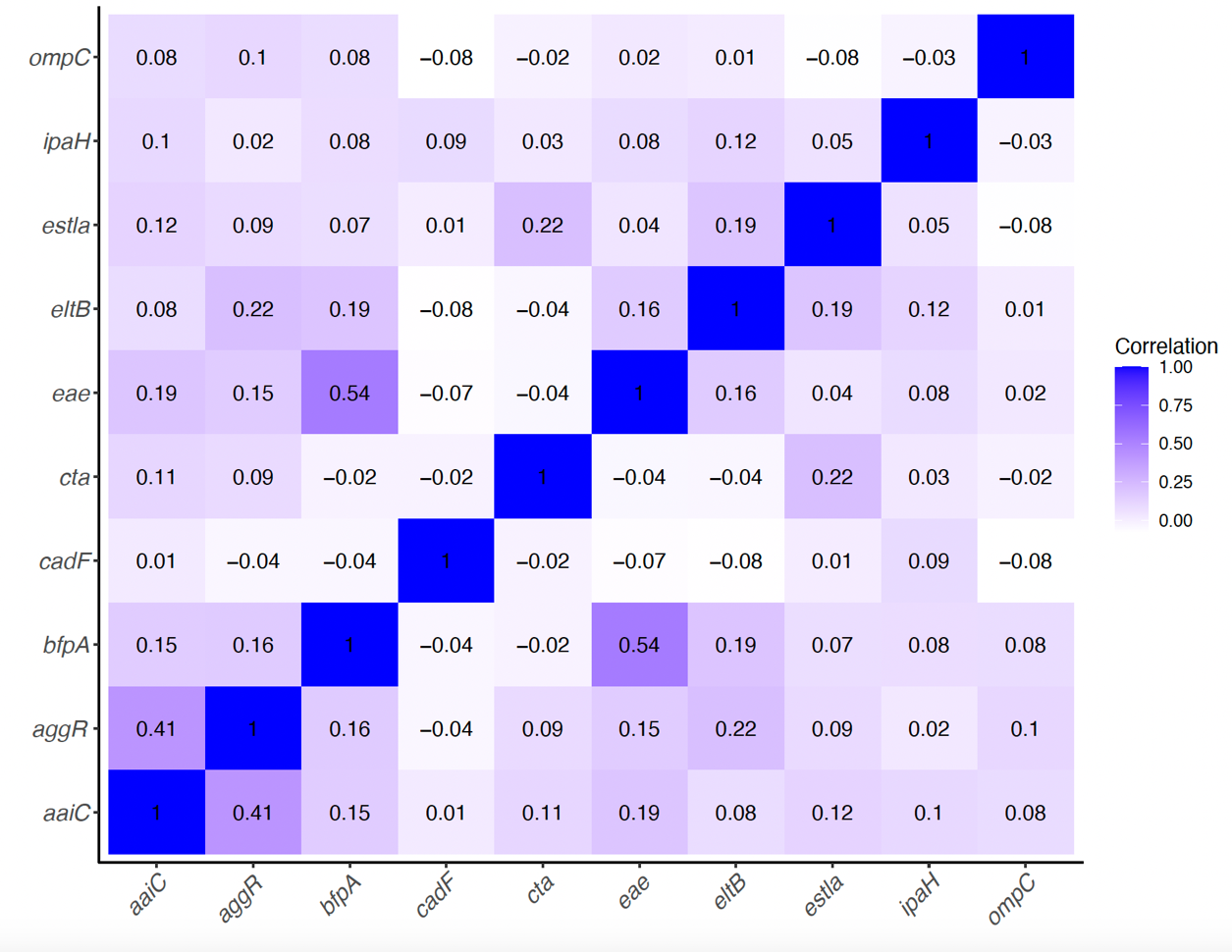
**

**F)**

**
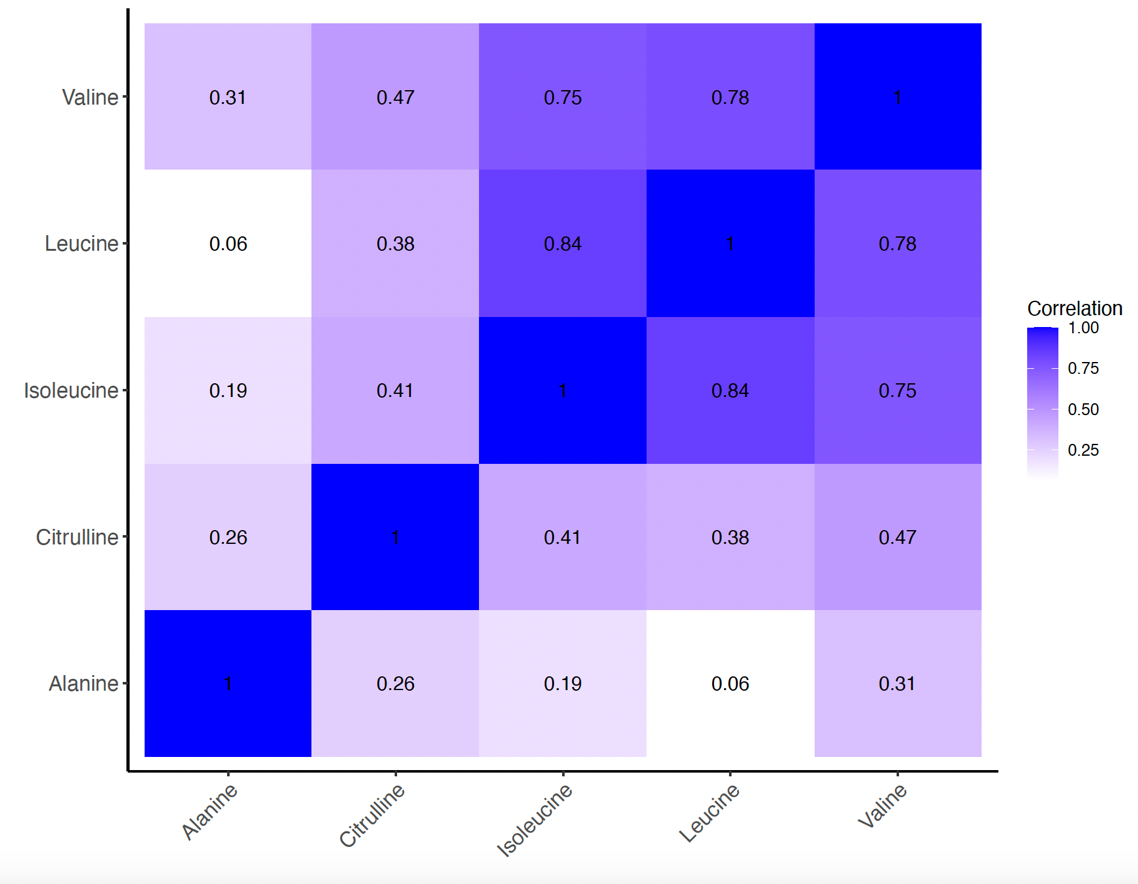
**

**SUPPL FIGURE 6.** Pairwise correlations among variables of the different blocks for the construction of latent variables: A) neurodevelopment, B) socio-economic status and maternal factors, C) inflammation, D) parasites and enteropathogens, E) enteropathogens’ genes, F) branched-chain amino acids.

If variables were continuous, we used Spearman’s r coefficient; if they were categorical, we used Cramer’s V statistic; if one was continuous and the other one categorical, we used the square root of the R^2^ of an ANOVA test.

CRP level was used as an inflammation marker, and considered elevated if above 10 mg/L. Citrulline was considered low if below 7 μmol/L and elevated if above 43 μmol/L. For AAT, values below 1.25 mg/g of fecal dry weight or below 0.15 of fecal wet weight were considered normal. For calprotectin, thresholds were adapted to age and were considered normal if the calprotectin levels were below 150 mg/g of fecal wet weight for children aged 2–3 years and 100 mg/g of fecal wet weights for children aged three years and older. Maternal education categories are: 1) None or primary school, 2) Middle school, 3) High school or more.

PS: Problem-Solving, PES: Personal-Social; FM: Fine Motor; GM: Gross Motor; Comm: Communication. AAT: α1-antitrypsin; CRP: C-reactive protein

**SUPPL FIGURE 7**. Mean direct effects (β-coefficients) of variables on neurodevelopment in the four different tested models: the simplest SEM, the simple SEM, the complex SEM and the complex path analysis. Significant associations (p-value ≤ 0.05) are indicated in red.

Units: Reported birth size categories: Smaller than other babies, same as other babies, bigger than other babies; Age in months; Maternal age at first pregnancy in years; α1-antitrypsin: normal vs elevated. For AAT, values below 1.25 mg/g of fecal dry weight or below 0.15 of fecal wet weight were considered normal.

PC: principal component; AAT: α1-antitrypsin.
